# Correlates of STI symptoms among female sex workers with truck driver clients in two Mexican border towns

**DOI:** 10.1186/1471-2458-12-1000

**Published:** 2012-11-20

**Authors:** Nadine E Chen, Steffanie A Strathdee, Felipe J Uribe-Salas, Thomas L Patterson, Maria Gudelia Rangel, Perth Rosen, Kimberly C Brouwer

**Affiliations:** 1 Department of Medicine, University of California San Diego, San Diego, CA, USA; 2 El Colegio de la Frontera Norte, Tijuana, Baja California, México; 3 Division of Global Public Health, University of California, San Diego; 9500 Gilman Dr, San Diego, CA, 92093-0507, USA

**Keywords:** Sexually transmitted infections, Sexually transmitted diseases, Substance use, Alcohol, Drugs, Female sex workers

## Abstract

**Background:**

Female sex workers (FSW) are at increased risk for HIV and other STI due to occupation-related risks and exposures. Long-distance truck drivers have been implicated in the spread of HIV, but less is known about HIV/STI risks of FSW servicing truck drivers, especially in North America. As part of an international collaborative pilot study, we interviewed FSWs servicing truck driver clients along two major transportation corridors to explore factors associated with recent STI symptoms.

**Methods:**

A cross-sectional study of 200 FSW was conducted in Mexico: 100 from Nuevo Laredo (U.S. border); 100 from Ciudad Hidalgo (Guatemalan border). Eligibility criteria included age ≥18 years, speaking English or Spanish, and having ≥1 truck driver client in the past month. The main outcome was reporting any recent STI symptoms, defined as experiencing genital/anal warts, genital ulcers/sores, genital itching, or abnormal vaginal discharge in the past 6 months. Logistic regression was used to identify correlates of recent STI symptoms.

**Results:**

Median age of FSW was 29 years, 74% were single, 87% had <9^th^ grade education, and median income was 4000 pesos/month ($300 USD). Sex work occurred at a bar/cantina for 70%. One-quarter had never been tested for HIV, 53% reported lifetime drug use, 22% reported drinking alcohol before/during transactional sex and 17% reported recent STI symptoms. After controlling for age and study site, factors associated with STI symptoms were lifetime drug use (AOR 2.9, 95% CI 1.2-6.9), drug use before/during sex (AOR 2.8, 95% CI 1.1-7.1), alcohol use before/during sex (AOR 5.2, 95% CI 2.2, 12.6), forced sex ever (AOR 2.6, 95% CI 1.1-6.1), lifetime history of arrest (AOR 2.3, 95% CI 1.0-5.0), and being surveyed in Nuevo Laredo rather than Ciudad Hidalgo (AOR 4.8, 95% CI 2.0-10.0).

**Conclusions:**

The associations we observed between recent STI symptoms and drug and alcohol use suggest that interventions are needed that promote consistent and effective safer sex practices, especially while under the influence of alcohol or other substances.

## Background

Female sex workers (FSW) are especially vulnerable to sexually transmitted infections (STIs) and HIV and have higher rates of infection than the general population [1-3]. Due to their occupation, FSW are often exposed to unprotected sex, sex with multiple partners, and substance use [3-5]. In addition, they are at risk for sexual and physical violence [3,5,6]. These factors increase vulnerability to STI/HIV infection.

Migration and mobility have been associated with the spread of HIV and other STIs, especially as mobility facilitates the mixing of sexual networks [7]. Long-distance truck drivers have been implicated in the early spread of HIV in Africa, India, and Asia [8-11]. Much less is known about the risk behaviors and STI/HIV prevalence of trucker drivers and their FSW acquaintances in the Americas, although reports suggest high transmission potential [12,13]. In 2002, a study of truck drivers along the Mexico-Guatemala border found that 26% reported sex with a FSW, and only 27% of those reported using condoms with a FSW [14]. In a U.S. study of truckers, one-third reported having frequent sexual intercourse on the road with multiple partners, but few reported condom use [15]. Substance use, especially alcohol use, has also been found to be common among truckers and is associated with unprotected sex [14,15]. Therefore, high risk behaviors among truckers coupled with their mobility and potential to transmit disease across regions in a short period of time, likely increase the STI/HIV risk to their sex partners.

The STI/HIV vulnerability of FSW servicing long-distance truckers in Mexico is unknown. Among FSW in Mexican-U.S. border cities, HIV prevalence was 17–26 times greater than that of the general Mexican population, and 36% of FSW had at least one bacterial STI [1,4]. We report results from an international collaborative pilot study evaluating truck drivers and FSW with truck driver clients. The objective of the present analysis was to identify factors associated with recent STI symptoms among FSW who service truckers in order to inform public health interventions to prevent the spread of HIV and other STIs.

## Methods

### Study population

Cross-sectional interviewer-administered surveys were conducted from August 2009 to January 2010 among 200 FSW (n = 100 in Nuevo Laredo at the Mexico/U.S. border, and n = 100 in Ciudad Hidalgo at the Mexico/Guatemala border). The Nuevo Laredo/Laredo border crossing is the largest truck freight crossing in North America while Ciudad Hidalgo is located on a major freight corridor between Mexico and Guatemala. Participants were selected through modified venue-based sampling [16], in which no more than 10 participants were enrolled from any particular site. FSW were eligible for the study if they were ≥18 years-old, spoke either English or Spanish, and worked at a truck stop or had at least 1 truck driver client in the past month.

### Data collection

Trained female staff members administered quantitative surveys to collect information on demographics, socioeconomic factors, working conditions, and access to care. Information pertaining to healthcare access was assessed by determining health insurance status, measuring number of gynecologic visit within the last year, and determining if participants had ever been tested for HIV. Deportation was assessed by self-report, and participants were asked from which country and to which country they were deported.

Substance use and sexual risk behaviors were by self-report. Frequency of alcohol use was dichotomized at several times a week versus once a week or less, as this was the median. Heavy alcohol use was defined as having >4 drinks, as four or more drinks within a two-hour period suggests binge drinking for women [17]. Participants were asked about lifetime use of illegal drugs in general and about specific substances, including marijuana, heroin, cocaine, and methamphetamines. Hard drug use was defined as cocaine, heroin, methamphetamine or amphetamine use. In addition, information about drug use in the past 6 months was collected. Information on sex work practices included place of sex work, average number of partners in the last six months, condom availability, client factors, and substance use before or during sex. Participants were considered to have been forced to have sex if they answered affirmative to the question “has anyone ever forced you to have sex with them using either physical force or emotional pressure”? Presence of a pimp or manager was assessed by asking whether participants had to currently pay someone “like a pimp, manager, or administrator or ….share with someone a percentage of the money” that they receive from clients. We also assessed nationality of clients as a previous study of Mexican FSW in border cities showed a greater prevalence of HIV and other STI among FSW with U.S. clients compared to those without U.S. clients [1].

The main variable of interest was recent STI symptoms, which we defined as having had genital/anal warts, genital ulcers or sores, genital itching, or abnormal vaginal discharge in the past 6 months. Participants were compensated $15 USD for their time. All participants provided informed written consent to participate in the study. The study was approved by the University of California at San Diego Human Research Protections Program and the Ethics Committee of El Colegio de la Frontera Norte in Tijuana, Mexico.

### Statistical analysis

The main outcome was recent STI symptoms. To compare differences by STI symptoms, we used Wilcoxon’s rank sum for continuous variables and Chi2 analysis for dichotomous variables. We then performed bivariate and multivariate logistic regression to evaluate independent factors associated with the outcome of recent STI symptoms. Only variables achieving a p < 0.10 on descriptive analysis were included in the bivariate analysis. In the multivariate model, we controlled for the demographic factors of age and study site. History of STI diagnosis ever was not included in the multivariate model due to collinearity with the outcome of interest. Missing data in multivariate logistic regression analysis was assigned the null value for conservative estimates.

## Results

The average FSW enrolled was single, a median age of 29 years-old (IQR 23–35), had less than a 9^th^ grade (*secundaria*) education, and earned 4000 pesos (approximately $300 USD) per month (Table 1). The majority of FSW was born in Mexico, but had been in the current city of residence for less than 5 years. FSW in this study had been in sex work for a median of almost 5 years and had approximately 35 clients in the last 6 months, but this number ranged from 5–350. One-quarter reported never having been tested for HIV, but of those who had been tested, 74% reported that they were tested for HIV within the last year (data not shown). Although most did not have health insurance, only 18% reported no regular access to healthcare, and 92% reported that they had had at least 1 gynecologic visit in the past year.

**Table 1:** Demographics and risk behavior characteristics of female sex workers servicing truck drivers in Mexico

**DEMOGRAPHICS**	**Total, No. (%),n=200**	**No STI symptoms, n=167**	**STI symptoms, n=33**	**p-value**
**Age** median years (IQR)	28.7 (22.8, 35.1)	28.8 (22.4, 35.1)	28.4 (24.7, 35.1)	0.43
**Civil status:**				
Married/common-law	52 (26.0%)	45 (26.9%) 122	7 (21.2%) 26	0.49
Single/divorced/separated/widow	148 (74.0%)	(73.1%)	(78.8%)	
**Education:**				
>9^th^ grade (*preparatoria)*	27 (13.5%)	21 (12.6%)	6(18.2%)	0.39
<9^th^ grade (*secundaria*)	173 (86.5%)	146 (87.4%)	27 (81.8%)	
**Income:** Monthly in thousand pesos (median, IQR)	4.0 (2.0, 10.0)	4.0 (2.0, 10.0)	5.3 (2.5, 12.0)	0.28
**Site**				
Nuevo Laredo	100 (50%)	74 (44.3%)	26 (78.8%)	<0.001
Ciudad Hidalgo	100 (50%)	93 (55.7%)	7 (21.2%)	
MIGRATION FACTORS				
**Birthplace:**				
Mexico	113 (56.5%)	87 (52.1%)	26 (78.8%)	0.005
Other Central American country	87 (43.5%)	80 (47.9%)	7 (21.2%)	
**Length of time in current city:**				
> 5 years	101 (50.8%)	80 (48.2%)	21 (63.6%)	0.11
< 5 years	98 (49.2%)	86 (51.8%)	12 (36.4%)	
**Ever been deported** (vs. never deported)	32 (16.0%)	26 (15.6%)	6 (18.2%)	0.71 ^a^
HEALTHCARE ACCESS				
**Health insurance,** last 6 months				
No	40 (20.0%)	30 (18.0%)	10 (30.3%)	0.11
Yes	160 (80.0%)	137 (82.0%)	23 (69.7%)	
**# Gynecologic visits,** last				
year(n=182)	14 (7.8%)	14 (9.1%)	0	0.090
0 >1	168 (92.3%)	139 (90.9%)	29 (100.0%)	
**Ever tested for HIV**				
No	51 (25.5%)	44 (26.3%)	7 (21.2%)	0.54
Yes	149 (74.5%)	123 (73.7%)	26 (78.8%)	
SUBSTANCE USE AND INCARCERATION				
**Heavy alcohol use:** Has (n=174)				
1-4 drinks on typical drinking day	30 (17.2%)	27 (18.2%)	3 (11.5%)	0.40
>4 drinks on typical drinking day	144 (82.8%)	121 (81.8%)	23 (88.5%)	
**Frequency of alcohol use **(n=189)				
Never-1x/week	58 (30.7%)	53 (33.3%)	5 (16.7%)	0.069
Several times/week- daily	131 (69.3%)	106 (66.7%)	25 (83.3%)	
**Lifetime drug use:**^b^				
Illicit drug use^c^	105 (52.5%)	81 (48.5%)	24 (72.7%)	0.011
Marijuana use	66 (33.2%)	55 (33.1%)	11 (33.3%)	0.98
Cocaine use	79 (39.5%)	62 (37.1%)	17 (51.5%)	0.12
Heroin use (n=199)	9 (4.5%)	5 (3.0%)	4 (12.1%)	0.021
Methamphetamine use	3 (1.5%)	2 (1.2%)	1 (3.0%)	0.43
Amphetamine use (n=197)	11 (5.6%)	8 (4.9%)	3 (9.1%)	0.34
Injection drug use (n=106)	7 (6.6%)	4 (4.9%)	3 (12.5%)	0.19
**Drug use in the last 6 months:**^b^				
Marijuana	25 (12.5%)	21 (12.6%)	4 (12.1%)	0.94
Cocaine	42 (21.0%)	33 (19.8%)	9 (27.8%)	0.33
Heroin	4 (2.0%)	3 (1.8%)	1 (3.0%)	0.64
Amphetamines	7 (3.5%)	5 (3.0%)	2 (6.1%)	0.38
**Ever arrested** (n=199)				
No	142 (71.4%)	124 (74.7%)	18 (54.6%)	0.019
Yes	57 (28.6%)	42 (25.3%)	15 (45.5%)	
**Arrested in the last year**				
No	167 (83.5%)	143 (85.7%)	24 (72.7%)	0.068
Yes	33 (16.5%)	24 (14.4%)	9 (27.3%)	
SEXUAL BEHAVIORS AND PRACTICES				
**Ever diagnosed with an STI**^d^				
No	172 (86.0%)	149 (89.2%)	23 (69.7%)	0.003
Yes	28 (14.0%)	18 (10.8%)	10 (30.3%)	
**Number of years worked as sex worker** (median, IQR)	4.90 (1.80, 18.0)	4.52 (1.78, 17.8)	7.07 (3.39, 23.3)	0.076
**Number of clients**, last 6 mo. (median, IQR) (n=196)	35 (10, 100)	30 (10,100)	50 (16.5, 174)	0.32
**Free condom availability**				
Never receives free condoms	80 (40.0%)	72 (43.1%)	8 (24.2%)	0.043
Receives sometimes/always	120 (60.0%)	95 (56.9%)	25 (75.8%)	
**Can you afford to buy own condoms?**				
No	21 (10.5%)	15 (9.0%)	6 (18.2%)	0.11
Yes	179 (89.5%)	152 (91.0%)	27 (81.8%)	
**Main place of sex work**				
Bar/cantina	140 (70.0%)	119 (71.2%)	21 (63.6%)	0.42
Other	60 (30%)	48 (28.8%)	12 (36.4%)	
**Has a manager/pimp**				
No	153 (76.5%)	124 (74.2%)	29 (87.9%)	0.092
Yes	47 (23.5%)	43 (25.8%)	4 (12.1%)	
**Alcohol before/during sex work,** last mo. (n=199)				
Never/sometimes/50% of the time	156 (78.4%)	138 (83.1%)	18 (54.5%)	<0.001
Often/always	43 (21.6%)	28 (16.9%)	15 (45.5%)	
**Used drugs before/during sex,** last mo.(n=198)				
Never	170 (85.9%)	148 (89.2%)	22 (68.7%)	0.002
Sometimes/often/always	28 (14.1%)	18 (10.8%)	10 (31.3%)	
**U.S. clients**, past 6 months				
No	138 (69.0%)	124 (74.2%)	14 (42.4%)	<0.001
Yes	62 (31.0%)	43 (25.8%)	19 (57.6%)	
**Has ever been forced to have sex** (n=199)				
No	161 (80.9%)	139 (83.7%)	22 (66.7%)	0.02
Yes	38 (19.1%)	27 (16.3%)	11 (33.3%)	
**Age** median years (IQR)	28.7 (22.8, 35.1)	28.8 (22.4, 35.1)	28.4 (24.7, 35.1)	0.43

^a^ Chi-2 p-value comparing ever deported vs. never deported.

^b^ All p-values are compared to null use.

^c^ Illicit drug use: includes heroin, cocaine, methamphetamine, inhalants, amphetamine, agua celeste, tranquilizers, barbiturates, ecstasy, and marijuana.

^d^ Variable was not evaluated in multivariate model due to collinearity with outcome.

In terms of substance use, 83% reported heavy alcohol use (more than four drinks on a typical drinking day) and 69% reported drinking alcoholic beverages several times per week. The majority reported ever using illicit drugs, including marijuana. Cocaine was the most commonly reported hard drug used in the last 6 months (n = 42). Alcohol use during sex transactions was common, with 22% reporting always or often using alcohol prior to or during sex work in the last month. Another 14% reported using drugs at least once prior to or during sex work in the last month.

A total of 17% (n = 33) reported having STI symptoms in the last six months, of whom 70% expressed >1 STI symptom. Only 14% of all FSW reported ever being formally diagnosed with an STI.

Table 2 shows the unadjusted and adjusted associations for recent STI symptoms. After controlling for age and study site, several different substance use factors remained significant. Lifetime history of drug use more than doubled odds of having recent STI symptoms (AOR 2.3, 95% CI 1.0-5.0), and drug use before or during sex work was an even stronger association with AOR 2.8 (95% CI 1.1, 7.1) having recent STI symptoms. Alcohol use before or during sex work was the most statistically significant association, increasing odds of having recent STI symptoms by 5.3 (95% CI 2.2-12.6). Other significant vulnerable factors included lifetime history of arrest and forced sex, both of which more than doubled odds of recent STI symptoms. FSW surveyed at the Mexico/Guatemala border site were 89% less likely to report STI symptoms than those from the Mexico/U.S. border site.

**Table 2:** Correlates of STI symptoms among FSW with truck driver clients in Mexico

**DEMOGRAPHICS**	**Unadjusted odds ratio**	**Adjusted odds ratio**^**a**^
**Age** median years (IQR)	1.02 (0.98, 1.06)	1.00 (0.96, 1.05)
**Site**		
Nuevo Laredo	Ref.	Ref.
Ciudad Hidalgo	**0.21 (0.09, 0.53)**	**0.21 (0.09, 0.53)**
MIGRATION FACTORS		
**Birthplace:**		
Other Central American Country	Ref.	Ref.
Mexico	**3.42 (1.41, 8.30)**	0.63 (0.09, 4.25)
HEALTHCARE ACCESS		
**# Gynecologic visits,** last year		
0	Ref.	
>1	1.46 (0.48, 4.48)	
SUBSTANCE USE AND INCARCERATION		
**Frequency of drinking**		
**alcohol **(n=189)		
Never-1x/week	Ref.	
Several times/week- daily	2.50 (0.91, 6.90)	
**Lifetime drug use:**		
Illicit drug use ever ^b^		
No	Ref.	Ref.
Yes	**2.83 (1.24, 6.45)**	**2.92 (1.24, 6.87)**
Hard drug use ever (excludes marijuana) ^c^		
No	Ref.	
Yes	2.02 (0.92, 4.47)	
Heroin use ever (n=199)		
No	Ref.	Ref.
Yes	**4.44 (1.13, 17.53)**	2.93 (0.70, 12.2)
**Ever arrested** (n=199)		
No	Ref.	Ref.
Yes	**2.46 (1.14, 5.31)**	**2.26 (1.02, 5.02)**
**Arrested in the last year**		
No	Ref.	
Yes	2.23 (0.93, 5.39)	
SEXUAL BEHAVIORS AND PRACTICES		
**Number of years worked as sex worker**	1.04 (0.99, 1.09)	
**Free condom availability**		
Never receives free condoms	Ref.	Ref.
Receives free condoms sometimes/always	**2.37 (1.01, 5.56)**	1.80 (0.74, 4.38)
**Has a manager/pimp**		
No	Ref.	
Yes	0.40 (0.13, 1.20)	
**Alcohol before/during sex work,** last mo. (n=199)		
Never/sometimes/ 50% of the time	Ref.	**Ref.**
Often/always	**4.11 (1.85, 9.11)**	**5.26 (2.20, 12.6)**
**Used drugs before/during sex work,** last mo.		
Never	Ref.	Ref.
Sometimes/50% time/often/always	**3.73 (1.53, 9.13)**	**2.82 (1.12, 7.10)**
**U.S. clients,** past 6 months		
No	Ref.	Ref.
Yes	**3.91 (1.81, 8.47)**	1.96 (0.73, 5.24)
**Has ever been forced to have sex**		
No	Ref.	Ref.
Yes	**2.57 (1.12, 5.92)**	**2.56 (1.07, 6.11)**
	**Unadjusted odds ratio**	**Adjusted odds ratio**^**a**^

^a^ Controlling for age and study site.

^b^ Illicit drug use: includes heroin, cocaine, methamphetamine, inhalants, amphetamine, agua celeste, tranquilizers, barbiturates, ecstasy, and marijuana.

^c^ Hard drug use: includes methamphetamine, heroin, cocaine, and amphetamines.

Abbreviations: *STI* sexually transmitted infection; *IQR* interquartile range; *mo* month.

## Discussion

In this study of FSW with trucker clients from two border towns in Mexico, although most FSW did not have health insurance, access to sexual and reproductive healthcare services did not seem to be an issue with the overwhelming majority having accessed gynecologic care and HIV testing within the last year. Despite access to these health services, approximately 1 out of 6 FSW reported recent STI symptoms, with substance use increasing odds of having STI symptoms. The prevalence of heavy alcohol use was common, but only FSW who reported frequently using alcohol within the context of sex work were more likely to report recent STI symptoms. By contrast, drug use at any time (both lifetime history of use and use in the context of transactional sex) was associated with recent STI symptoms. Factors that may indicate increased marginalization from society, including arrest history and history of forced sex, were also associated with recent STI symptoms. In addition, FSW who were surveyed in Ciudad Hidalgo were less likely than those from Nuevo Laredo to report recent STI symptoms.

The link between alcohol and STI/HIV risk has been well established [18]. Several mechanisms have been studied for this association, including behavioral disinhibition while under the influence and the predisposition of a sensation-seeking personality which leads to both increased sexual risks as well as increased alcohol use [18,19]. Previous studies, not restricted to FSW, have shown that generalized alcohol consumption, as well as consuming alcohol prior to or at the time of sexual relations, increases sexual risk behaviors and risk of HIV acquisition [20,21]. In this study, while heavy alcohol consumption was not associated with increased STI risk, drinking prior to or during sex work was associated with increased STI risk. The majority of FSW reported their main place of sex work occurred at bars or cantinas where alcohol use is common, if not expected. For FSW hired by the bar or cantina, male patrons often must buy “drinks” for or from the FSW for the opportunity to spend time with them [22]. For FSW who are not employed by the establishment, soliciting clients at bars likely involves sharing drinks together. In several South African studies, drinking alcohol before sex or meeting a sex partner at a drinking establishment has been associated with unprotected sex acts [23-25]. Because bars were a common place to solicit clients, workplace interventions to improve safer sex practices while under the influence of alcohol, such as increasing condom availability in bar settings [26], may help decrease the spread of STIs.

In regards to drug use, both lifetime illicit drug use and drug use before or during sex work were associated with increased report of recent STI symptoms. In a previous study of FSW along Mexican-U.S. border towns, drug use prior to or during sex has been associated with increased risk of STI [2]. Recent cocaine and methamphetamine use (past month) has also been associated with HIV infection among Mexican FSW [27]. In this pilot study, although several FSW reported recent drug use, namely cocaine and marijuana, neither of these drugs were specifically associated with recent STI symptoms. This is likely due to the small sample size precluding ability to detect a difference. As with alcohol, drug intoxication prior to sex can decrease ability to negotiate condom use and thereby increases STI risk. Cocaine, especially crack cocaine, has been associated with increased STI rates [28]. As cocaine was the most common drug used in our study sample, an intervention to increase condom use among drug users, particularly those who use cocaine, needs to be further explored.

Besides substance use, violence or threat of violence against FSW increases STI/HIV vulnerability, largely through decreased ability to negotiate condom use [5,29]. In this study, history of ever being forced (through either physical or emotional pressure) to have sex was significantly associated with recent STI symptoms. Several studies have shown that women in violent relationships are less likely to negotiate condom use or to refuse sex by HIV-infected partners [30-32]. This violence can also have long-term emotional or psychological effects, such as decreased self-image and post-traumatic stress disorder and anxiety [29,33]. Among HIV-infected women, a history of abuse has been associated with decreased HIV medication use which was attributed to decreased self-care [33]. Safer work environments that help reduce sexual violence against FSW have been associated with empowerment of FSW for both the improvement of their working conditions as well as ability to negotiate condom use [22,34,35].

Similarly the criminalization of prostitution or harassment of FSW by police may drive FSW to less safe places, which may increase their risk of violence and impact safer sex practices. One study of Canadian FSW found that those FSW who were working away from main streets because of policing were three times more likely to have unprotected sexual intercourse [5]. In our study, FSW who have ever been arrested were more likely to report recent STI symptoms. Women may be less likely to seek help from law enforcement when assaulted if they have had a previous negative interaction with police. In addition, history of arrest could indicate other risky behaviors, such as drug use, that also increase risk of STI. The convergence of substance use, violence, and HIV/AIDS epidemics, also known as the SAVA syndemic, has been found to be especially prevalent among women within the criminal justice system in the United States [36]. An understanding of the reasons for the association of recent STI symptoms with a history of arrest needs to be further explored as it could indicate either an environmental barrier from harassment by police versus an individual level vulnerability from other factors related to substance use, mental health, and violence as described by the SAVA syndemic.

Interestingly, FSW recruited from Ciudad Hidalgo were less likely to report recent STI symptoms than FSW from Nuevo Laredo. Although reasons for this difference are unclear, a possible explanation may include sexual network differences in STI/HIV prevalence, as one study of FSW along the Mexico-U.S. border showed that FSW with U.S. clients were more likely to have STIs [1]. Due to geographic proximity, FSW in Nuevo Laredo were more likely to have U.S. clients than those from Ciudad Hidalgo. Public health interventions such as STI/HIV screening, safer-sex education, and condom promotion may also differ by area, and these differences likely influence risk behaviors. In our sample, although FSW from Nuevo Laredo were more likely to report access to regular healthcare and to have health insurance than those from Ciudad Hidalgo, FSW at the southern border were more likely to have ever been HIV tested and score higher on the condom efficacy scale (data not shown). Differences in consistent condom use by region may be affected by several different factors including condom availability, peer norms regarding condom use, and public health programs to promote condom use. Thus, not only is access to care important, but effective public health programs to promote HIV/STI prevention and screening are necessary.

There are several limitations that should be considered in the interpretation of our data. As this was a cross-sectional study, we were unable to draw any causal inferences. In addition, this was a pilot study with a relatively small sample size, and we therefore may have been underpowered to detect some associations (e.g. specific drug use and drugs used in the last 6 months). Similar to other studies of sexual behaviors and substance use, social desirability may have limited self-report of risk behaviors. In this pilot study, presence of STI symptoms were self-reported and has not been validated as a proxy measure for actual STI prevalence. Certain symptoms which were attributed to being from an STI (e.g. genital itching or abnormal vaginal discharge) could also be caused by non-STI related diseases, such as vaginal candidiasis or bacterial vaginosis. Therefore, self-assessment can overestimate actual STI prevalence. On the other hand, some women may be asymptomatic or not recognize symptoms of certain STI (e.g. HIV, syphilis, and chlamydia), especially in the early stages, thereby underestimating STI prevalence. Lastly, this study only involved FSW servicing truck driver clients and therefore the findings may not be generalizable to FSW in the region without truck driver clients.

## Conclusion

Among FSW in Mexico who have truck driver clients, substance use was strongly associated with increased report of STI symptoms. STI/HIV prevention efforts need to address safer sex practices while under the influence of alcohol or drugs. As a majority of the sex work occurred in bars, increased condom availability in bars may be a simple yet effective intervention to increase condom use among FSW [26]. The association history of arrest and/or forced sex with increased STI symptoms may be linked to the SAVA syndemic. Thus providing safe spaces for sex work may help decrease the violence against FSW and empower condom use to decrease STI risk.

## Competing interests

The authors declare that they have no completing interests.

## Authors’ contributions

All authors listed made substantial intellectual contributions to this manuscript. GR, PR, FUS, and KCB made substantial contributions to the design of the parent study and acquisition of data. NEC, KCB, TLP, and SAS contributed to the conception and interpretation of the data. NEC performed the statistical analysis and was responsible for primary draft of the manuscript. All authors contributed to and have read and approved the final version of the manuscript.

## Pre-publication history

The pre-publication history for this paper can be accessed here:

http://www.biomedcentral.com/1471-2458/12/1000/prepub
